# Systemic Mastocytosis With Associated Acute Myeloid Leukemia Identified After Induction Therapy: A Case Report and Literature Review

**DOI:** 10.7759/cureus.116677

**Published:** 2026-09-21

**Authors:** Joseph Han, Farid Ghamsari, Jessica Yan, Phyllis Kim

**Affiliations:** 1 Internal Medicine, Olive View University of California Los Angeles Medical Center, Sylmar, USA; 2 Hematology and Oncology, Olive View University of California Los Angeles Medical Center, Sylmar, USA

**Keywords:** acute myeloid leukemia (aml), allogeneic stem cell transplant (allo-sct), bone marrow biopsy, kit mutation, mast cell, systemic mastocytosis with an associated hematological neoplasm (sm-ahn)

## Abstract

Systemic mastocytosis (SM) is a rare clonal mast cell disorder, with the vast majority of cases driven by activating KIT mutations, that can coexist with other myeloid hematologic malignancies. There are multiple subtypes of SM, one of which is SM with an associated hematologic neoplasm (SM-AHN), which is the coexistence of SM and a separate neoplasm, often driven by a shared clonal origin. We report the case of a 48-year-old man who initially presented with profound leukocytosis, anemia, and thrombocytopenia and was subsequently diagnosed with AML. After undergoing induction chemotherapy, the patient was found to have less than 5% blasts but perivascular clusters of CD25-positive and CD117-positive cells on a day-14 bone marrow biopsy that were not previously seen, establishing the diagnosis of SM-AML. The patient is currently undergoing consolidation with consideration of allogeneic stem cell transplantation and potential additional maintenance therapy for SM. SM-AHN is a rare entity that has been reported in the literature; however, given its rarity, it does not have a well-established understanding of the drivers of malignancy, risk stratification, and prognostication, among other aspects that are better understood in more common malignancies. Here, we present a case of SM-AHN to contribute to the current literature and improve understanding of this rare entity.

## Introduction

Systemic mastocytosis (SM) is a clonal hematopoietic disease characterized by the abnormal proliferation of neoplastic mast cells in at least one extracutaneous organ [1]. Per WHO criteria, the diagnosis of SM requires either (1) one major criterion plus one minor criterion, or (2) three minor criteria [1,2]. The major criterion is evidence of multifocal dense infiltrates of mast cells detected in sections of bone marrow and/or other extracutaneous organ(s) [2]. The four minor criteria include: (1) atypical mast cell morphology present in >25% of all mast cells on bone marrow smears or in other extracutaneous organ(s); (2) mast cells aberrantly expressing one or more of the following antigens: CD2, CD25, or CD30; (3) a KIT p.D816V mutation or other activating KIT mutation detected in peripheral blood, bone marrow, or other extracutaneous organ(s); and (4) a baseline serum tryptase concentration of >20 ng/mL in the absence of an associated myeloid neoplasm (AMN) [2]. SM is classified into several subtypes: indolent SM (ISM), smoldering SM (SSM), SM with an associated hematologic neoplasm (SM-AHN), aggressive SM (ASM), and mast cell leukemia (MCL), the diagnosis of which is partly informed by the presence of B-findings, reflecting mast cell burden, and C-findings, reflecting end-organ dysfunction, in addition to the presence of an AHN [1]. Ultimately, a significant decision point in management is determining whether a patient has either an AHN or C-findings, which help determine the need for cytoreductive therapy [1,2].

SM is rare, with an estimated prevalence of 0.46 per 100,000 people in the United States; however, up to 40% of patients with advanced SM (SM-AHN, ASM, and MCL) have an associated hematologic neoplasm [3,4]. Among SM-AHN cases, acute myeloid leukemia (AML) is uncommon, with one study reporting that AML accounts for approximately 3% of SM-AHN cases [5]. SM-AHN confers a worse prognosis than advanced overall SM, with one study reporting a median overall survival (OS) of 24 months versus 41 months [6]. Among SM-AHN subtypes, AML is associated with the poorest outcomes, with an average OS of 11 months [2]. Given the rarity of SM-AML, clinicians can benefit from individual case studies to help elucidate a condition that lacks well-established risk stratification, tailored treatment algorithms, and prognostication.

## Case presentation

We present the case of a 48-year-old man with no significant past medical history who initially presented to urgent care with two days of continuous, non-radiating epigastric abdominal pain. Vital signs were notable only for tachycardia, while physical examination was otherwise unremarkable. Notably, the patient did not have any symptoms or physical examination findings consistent with excess mast cell burden, such as flushing, anaphylaxis, or rash. Initial laboratory evaluation revealed leukocytosis, anemia, and thrombocytopenia, raising concern for a hematologic malignancy (Table 1). The patient was advised to present to the emergency department but deferred further evaluation. Flow cytometry of the initial peripheral blood sample showed findings consistent with AML five days later.

**Table 1:** Initial laboratory findings at presentation.

Laboratory test	Initial presentation	Reference range
White blood count (cells/L)	29.1 × 10⁹	4.5-10 x 10⁹
Percent neutrophils	14%	40-70%
Percent lymphocytes	19%	20-40%
Percent monocytes	36%	2-8%
Percent blasts	28%	0%
Hemoglobin (g/dL)	11.6	13.5-16.5
Platelet count (cells/L)	9 x 10⁹	160-360 x 10⁹

The patient re-presented to urgent care about three weeks later after being notified of the pathology results, now reporting fatigue, dyspnea on exertion, and a five-pound weight loss over the preceding weeks. He also noted mild gum bleeding but denied significant bruising, headache, blurry vision, or neurologic symptoms. On exam, the patient was noted to have right axillary edema and erythema with a 2-cm subcutaneous mass. The patient was referred to the emergency department, where a bone marrow biopsy confirmed AML. Molecular testing revealed a KIT exon 17 mutation (R815_D816delinsKI) with a variant allele frequency (VAF) of 41.8% and an ATM variant with a VAF of 48.3%. Cytogenetic analysis demonstrated a normal karyotype. His right axillary mass was also biopsied, showing leukemic infiltration of the skin (leukemia cutis), a finding associated with an adverse prognosis in AML. The patient was admitted for induction chemotherapy with standard 7+3 (cytarabine plus anthracycline). A day 14 bone marrow biopsy revealed perivascular clusters of CD25-positive, CD117-positive, CD2-negative, and CD30-negative, morphologically round mast cells that were not previously seen, consistent with SM, establishing the diagnosis of SM-AML (Figure 1). These mast cells were thought to have been obscured by the large population of blasts in the initial bone marrow. Of note, on re-review of the pathology of the right axillary mass, there was no infiltration of mast cells within the skin biopsy. At this time, a serum tryptase level was obtained, which was 81.6 mcg/L (reference range < 11.0 mcg/L). The patient completed induction therapy, and a day 35 bone marrow biopsy demonstrated a persistent ATM mutation, which was likely germline given the previously mentioned allele frequency, though confirmatory testing in non-hematopoietic tissue was not performed. Currently, the patient is undergoing consolidation therapy, which has been complicated by an admission for neutropenic fever and septic shock; however, he has since recovered and is continuing treatment. The patient has also been referred to an outside hospital for allogeneic stem cell transplantation with consideration of maintenance therapy.

**Figure 1:**
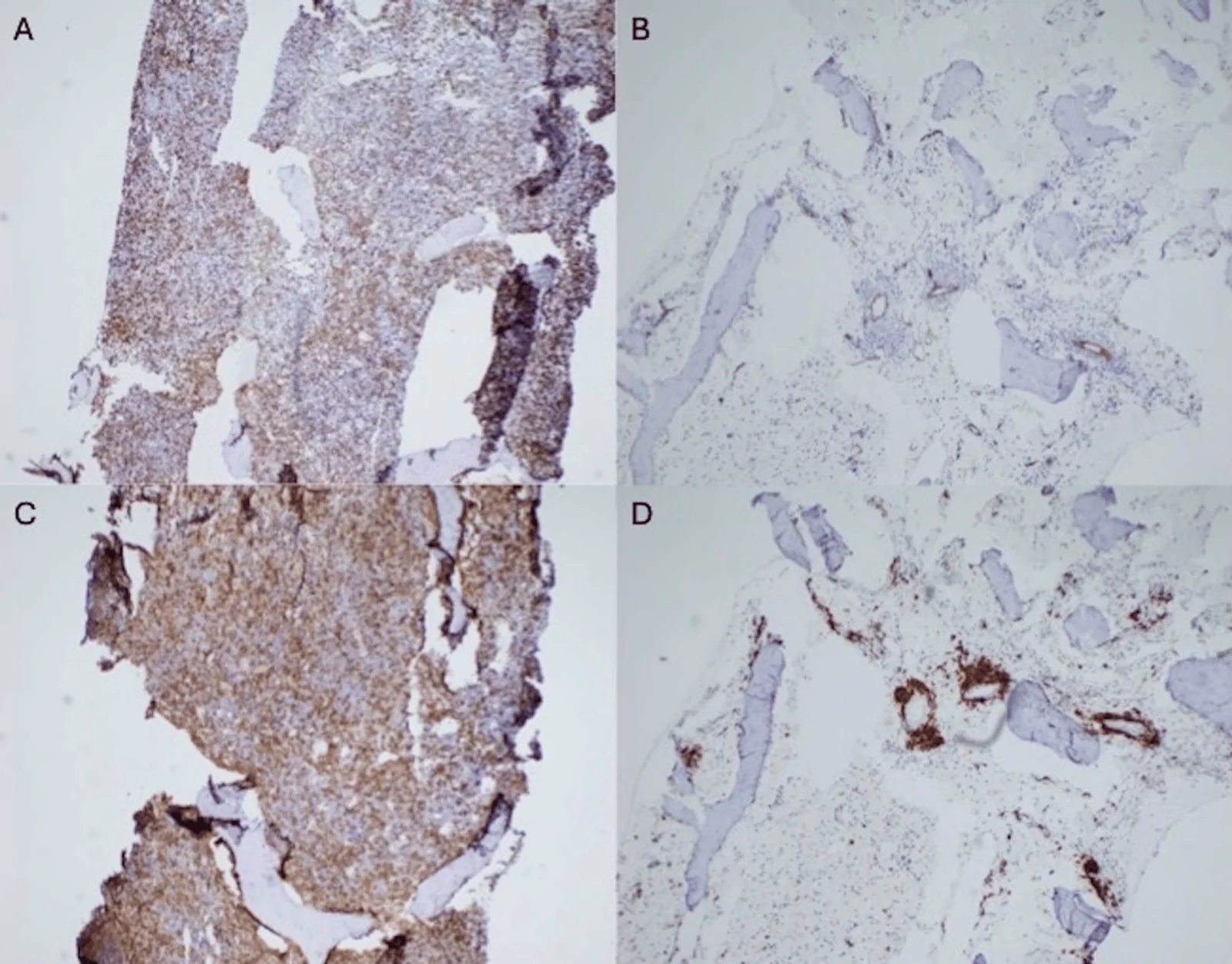
Pre-induction CD34 and CD117 staining (A, C) and post-induction day 14 CD34 and CD117 staining (B, D), respectively. Notably, panel D demonstrates perivascular clusters of CD117+ mast cells, which fulfill the major criterion.

## Discussion

This case illustrates a patient initially diagnosed with AML who was subsequently found to have SM following induction therapy. The diagnosis of SM requires fulfillment of the WHO major and minor criteria (one major plus one minor, or three minor criteria) through histological, immunohistochemical, biochemical, and molecular analyses (Table 2) [2]. SM can be further classified into subtypes based on the presence of an AHN and/or B/C findings (Table 3) [2]. This patient's bone marrow section fulfills the major criterion and additionally fulfills two minor criteria through the detection of an activating KIT mutation and aberrant expression of CD25 on mast cells.

**Table 2:** Major and minor diagnostic criteria for systemic mastocytosis.

Major criteria	Minor criteria
Multifocal dense infiltrates of mast cells (≥15 mast cells in aggregates) detected in sections of bone marrow and/or other extracutaneous organ(s).	Atypical mast cell morphology, including spindle shape or immature morphology, is present in >25% of all mast cells on bone marrow smears or in other extracutaneous organ(s).
	Mast cells aberrantly express one or more of the following antigens: CD2, CD25, and CD30.
	A KIT p.D816V mutation or other activating KIT mutation detected in peripheral blood, bone marrow, or other extracutaneous organ(s).
	Baseline serum tryptase concentration of >20 ng/mL in the absence of an AMN. In the case of a known hereditary alpha tryptasemia, the tryptase level could be adjusted.

**Table 3:** B-findings and C-findings of systemic mastocytosis.

B findings	C findings
High mast cell burden: Infiltration in bone marrow ≥30% and/or serum total tryptase ≥200 ng/mL and/or KIT p.D816V variant allele frequency ≥10% in bone marrow or peripheral blood leukocytes	≥1 cytopenia(s) found: absolute neutrophil count <1 × 10⁹/L, hemoglobin <10 g/dL, and/or platelet count <100 × 10⁹/L
Signs of myeloproliferation and/or myelodysplasia not fulfilling criteria for AHN	Hepatopathy: Ascites and elevated liver enzymes + hepatomegaly or cirrhotic liver + portal hypertension
Hepatomegaly on palpation or imaging (ultrasound, CT, or MRI) without ascites or other signs of organ damage and/or splenomegaly on palpation or imaging without hypersplenism and/or lymphadenopathy on palpation or imaging (>20 mm)	Spleen: Palpable splenomegaly with hypersplenism ± weight loss ± hypoalbuminemia
	Gastrointestinal tract: Malabsorption with hypoalbuminemia ± weight loss
	Bone: Large-sized osteolysis (≥20 mm) ± pathologic fracture ± bone pain

SM-AML represents a rare entity in which an activating KIT mutation (such as KIT R815_D816delinsKI, as in our patient) can affect multiple myeloid lineages, suggesting a shared clonal origin of both the mast cell and leukemic components [1]. Though the activating KIT variant is classically D816V, the D816I mutation identified in our patient has previously demonstrated activating effects on KIT signaling [7,8]. Importantly, the same overall mutational profile identified in our patient, including the additional R815K mutation, demonstrates an activating effect as well [9].

In cases like that of this patient, the mast cell proliferation can be masked by the associated AML, with SM only recognized retrospectively after cytoreduction reduces the leukemic burden, a phenomenon that has been reported in the literature [10]. Additionally, it is often the case that the symptoms of the AML overshadow any manifestations of the SM, such as flushing, hypotension, or bone pain, making the detection and diagnosis of the underlying SM even more difficult. Serum tryptase is also unreliable in the setting of concurrent AMN, which is why it is not considered a diagnostic minor criterion when there is an associated AMN [2]. SM-AML typically harbors at least one additional somatic mutation beyond KIT D816V [11]. In this case, a concurrent, likely germline, ATM pathogenic variant was also identified, and whether additional germline mutations influence disease progression and risk stratification in this context is unclear and warrants further investigation.

Given that the SM component was not readily apparent on initial evaluation in the case presentation, it is worth exploring how clinicians could potentially evaluate for underlying SM in cases like this where it was not clinically apparent. In one small study of 49 patients with myeloid neoplasms and associated KIT mutations, 8% qualified for a diagnosis of SM [12]. Another retrospective study evaluated 40 patients with KIT (D816V)-positive/core binding factor (CBF)-negative AML and found that all of them met diagnostic criteria for SM [11]. This prompted a retrospective analysis using a different database of patients with KIT-positive/CBF-negative AML. Although they could not confirm co-existing SM in that study group, both groups had strong similarities in allele frequency, mutation profile, and karyotype, suggesting that a large proportion of the second group likely had undiagnosed concurrent SM. A separate study showed that in a small cohort of patients with KIT-positive myeloid neoplasms, 91% of patients with myelodysplastic, myeloproliferative, or overlapping MDS/MPN who harbored KIT mutations also had SM, while 44% of patients with KIT-positive AML were found to have SM. Notably, in this study, one-third of patients with SM-AHN did not have their SM component recognized during the course of their disease [13]. Given these studies, there appears to be a strong association between KIT-positive AML and SM, particularly in CBF-negative AML. In our case, the patient was CBF-negative based on a normal karyotype, and the presence of KIT-positive AML could have warranted evaluation for SM given this strong association. This represents an interesting avenue for future research, particularly with respect to investigating whether certain cytogenetic and mutation profiles can warrant evaluation for SM in the setting of an AML diagnosis.

Management of SM-AML requires addressing both disease components; however, per NCCN guidelines, one process is usually addressed first based on severity [2]. Standard AML therapy, including high-dose induction chemotherapy, remains the cornerstone for achieving remission; however, due to the poor prognosis of SM-AML, allogeneic stem cell transplant (allo-SCT) is generally recommended for eligible patients [2]. The SM component may be managed with targeted KIT inhibitors such as avapritinib, midostaurin, or dasatinib, either to actively manage the SM component or as maintenance post-transplant [1]. However, clinicians should maintain vigilance for rare complications during targeted therapy, as illustrated by a reported case of fatal spontaneous splenic rupture in a CML patient on imatinib, in which non-specific abdominal pain was initially overlooked [14]. Prognostically, KIT-mutated SM-AML carries a poor outcome, with reported median overall survival (OS) as low as 5.4 months. Intensive chemotherapy with or without allo-SCT improves OS to approximately 16.7 months compared to 2.7 months with non-intensive therapy [11]. Individual reports have demonstrated successful remission using standard induction plus allo-SCT with or without concurrent KIT inhibition [15]. However, there is still relatively sparse literature on the best treatment approach for SM-AML.

One potential avenue is a treatment approach that addresses both the SM and AML components concurrently. Though no prospective trials exist to help guide the best treatment approach, there is literature that provides grounds for further investigation. For example, one case report of a patient with SM-AML who was initially treated with a hypomethylating agent (HMA) with poor response and was subsequently treated with a cycle of decitabine and venetoclax, achieving complete remission of both the SM and AML components [16]. Although bone marrow mast cells typically do not express BCL-2 to an appreciable degree, AML cells demonstrate strong BCL-2 expression, and in the setting of a pathology where both disease processes share a common progenitor, mast cells may express and respond to BCL-2 agents more robustly. Individual reports such as these may provide grounds for further investigation into more robust treatment regimens that may concurrently and more effectively address the disease and potentially improve outcomes.

These studies provide valuable insight into the potential role of KIT mutations and additional genotypes in prompting evaluation for underlying SM, particularly in the setting of myeloid neoplasms, as well as informing potential treatment approaches. Based on the referenced literature, the patient in the case presentation may have had more expedited identification of the underlying SM given the strong association of KIT-activated, CBF-negative AML with concomitant SM. Given the rarity of SM and its associated subtypes, and the lack of overt clinical manifestations of SM in a large proportion of cases, predictive features such as genotype may prove useful to clinicians in the future. Additionally, though sparse, there appears to be some literature that provides insight into potential avenues for novel treatment approaches. Ultimately, continued advances in our understanding of SM-AML may lead to improved diagnostic and therapeutic strategies and, hopefully, better outcomes for patients with SM-AML, which currently carries a poor prognosis.

## Conclusions

SM-AML is a rare entity that lacks a robust diagnostic and therapeutic framework, largely due to its low incidence and limited clinical experience. Genetic testing for KIT mutations in AML patients may help identify those at risk for concurrent SM and should be considered as part of the diagnostic workup. As a result, it may be underrecognized or misclassified until its full clinicopathologic spectrum becomes apparent, potentially leading to delayed diagnosis or failure to identify the underlying dual pathology. Through this case, we aim to contribute to the growing but still limited body of literature on SM-AML, highlight key features that may facilitate earlier recognition, and emphasize the importance of considering this diagnosis in appropriate clinical contexts. Improved awareness and characterization of this entity may support earlier diagnosis and the development of more effective, targeted therapeutic strategies that warrant further investigation.

## References

[1] Valent P, Sperr WR, Akin C (2010). How I treat patients with advanced systemic mastocytosis. Blood.

[2] (2025). NCCN Clinical Practice Guidelines in Oncology (NCCN Guidelines®): Systemic Mastocytosis. Version 2.2026. National Comprehensive Cancer Network.

[3] Bista A, Uprety D, Vallatharasu Y, Arjyal L, Ghimire S, Giri M, Rosenstein L (2018). Systemic mastocytosis in United States: a population-based study. Blood.

[4] Zhrebker L, Cooper B, Krause JR (2014). Systemic mastocytosis with associated acute myelogenous leukemia. Proc (Bayl Univ Med Cent).

[5] Pardanani A, Lim KH, Lasho TL, Finke C, McClure RF, Li CY, Tefferi A (2009). Prognostically relevant breakdown of 123 patients with systemic mastocytosis associated with other myeloid malignancies. Blood.

[6] Lim KH, Tefferi A, Lasho TL (2009). Systemic mastocytosis in 342 consecutive adults: survival studies and prognostic factors. Blood.

[7] Bodemer C, Hermine O, Palmérini F (2010). Pediatric mastocytosis is a clonal disease associated with D816V and other activating c-KIT mutations. J Invest Dermatol.

[8] Yang Y, Létard S, Borge L (2010). Pediatric mastocytosis-associated KIT extracellular domain mutations exhibit different functional and signaling properties compared with KIT-phosphotransferase domain mutations. Blood.

[9] Couch T, Murphy Z, Getman M, Kurita R, Nakamura Y, Steiner LA (2019). Human erythroblasts with c-Kit activating mutations have reduced cell culture costs and remain capable of terminal maturation. Exp Hematol.

[10] Srinivasan T, Manimuthu S, Jamwal M (2025). Post-induction bone marrow uncovers mastocytosis in a case of acute myeloid leukemia - case report. Curr Probl Cancer.

[11] Jawhar M, Döhner K, Kreil S (2019). KIT D816 mutated/CBF-negative acute myeloid leukemia: a poor-risk subtype associated with systemic mastocytosis. Leukemia.

[12] Kim DH, Jia F, Patel KP, Bose P, Wang SA, Bueso-Ramos C, Ok CY (2024). Utility of KIT mutations in myeloid neoplasms without documented systemic mastocytosis to detect hidden mast cells in bone marrow. Clin Lymphoma Myeloma Leuk.

[13] Craig JW, Hasserjian RP, Kim AS (2020). Detection of the KIT(D816V) mutation in myelodysplastic and/or myeloproliferative neoplasms and acute myeloid leukemia with myelodysplasia-related changes predicts concurrent systemic mastocytosis. Mod Pathol.

[14] Kanani J, Sheikh MI (2024). An autopsy presentation of spontaneous splenic rupture in chronic myeloid leukemia: a rare case report. J Med Surg Public Health.

[15] Zhou M, Zhong G, Yu Y, Wang R, Ma S, Chen C (2023). Successful treatment of systemic mastocytosis with acute myeloid leukemia: a case report. Ann Hematol.

[16] Pourhassan H, Kim Y, Pullarkat V (2023). Clearance of bone marrow mast cells after hypomethylating agent and venetoclax for systemic mastocytosis associated with myeloid neoplasia. Eur J Haematol.

